# Developmental and Early Life Origins of Hypertension: Preventive Aspects of Melatonin

**DOI:** 10.3390/antiox11050924

**Published:** 2022-05-08

**Authors:** You-Lin Tain, Chien-Ning Hsu

**Affiliations:** 1 Department of Pediatrics, Kaohsiung Chang Gung Memorial Hospital and Chang Gung University College of Medicine, Kaohsiung 833, Taiwan; tainyl@cgmh.org.tw; 2 Institute for Translational Research in Biomedicine, Kaohsiung Chang Gung Memorial Hospital, Kaohsiung 833, Taiwan; 3 Department of Pharmacy, Kaohsiung Chang Gung Memorial Hospital, Kaohsiung 833, Taiwan; 4 School of Pharmacy, Kaohsiung Medical University, Kaohsiung 807, Taiwan

**Keywords:** developmental origins of health and disease (DOHaD), glucocorticoid, gut microbiota, melatonin, hypertension, renin–angiotensin system, nitric oxide, oxidative stress

## Abstract

Hypertension represents a major disease burden worldwide. Abundant evidence suggests that hypertension can originate in early life. Adverse programming processes can be prevented by early life intervention—namely, reprogramming—to avoid developing chronic diseases later in life. Melatonin is an endogenously produced hormone with a multifaceted biological function. Although melatonin supplementation has shown benefits for human health, less attention has been paid to exploring its reprogramming effects on the early life origins of hypertension. In this review, first, we discuss the physiological roles of melatonin in pregnancy, fetal development, and the regulation of blood pressure. Then, we summarize the epidemiological and experimental evidence for the early life origins of hypertension. This is followed by a description of the animal models used to examine early melatonin therapy as a reprogramming strategy to protect against the early life origins of hypertension. A deeper understanding of the developmental programming of hypertension and recent advances in early melatonin intervention might provide a path forward in reducing the global burden of hypertension.

## 1. Introduction

Melatonin is an endogenous hormone that is mainly released by the pineal gland during the night [1,2]. Melatonin has a multifaceted biological function. It is capable of controlling circadian rhythms, redox homeostasis, intestinal motility, mitochondrial biogenesis, fetal development, etc. [3,4,5]. Melatonin, together with its metabolites, has antioxidant effects that make this molecule an endogenous protector against many oxidative stress-related disorders [6].

Hypertension is highly prevalent around the world, despite the tremendous advances made in healthcare and medicine [7]. Blood pressure (BP) shows multifactorial inheritance patterns. Hypertension is determined by the interaction of environmental factors and multiple genes, although genome-wide association studies merely explain ~3.5% of BP trait variability [8]. Indeed, human and animal studies indicate that hypertension can be programmed in early life [9,10,11,12,13]. The association between early life environmental insult stimuli and an increased susceptibility to adult disease has emerged as the theory of the developmental origins of health and disease (DOHaD) [14]. Notably, adverse programming processes can be reversed by early intervention, namely reprogramming, to avoid the development of chronic diseases throughout life [12,15].

During pregnancy, melatonin can pass the placenta or is produced by the placenta to transfer circadian signals to the fetus, which is essential for fetal development [16,17]. Published data support the notion that maternal treatment with melatonin can counteract the early life insult-induced programming process and thereby prevent the development of chronic diseases later in life [18]. Though melatonin has shown benefits for hypertension, the literature focusing on the use of melatonin as an early intervention to prevent the early life origins of hypertension remains limited.

To achieve the goal of this scoping review, a search was executed in electronic bibliographic database PubMed/MEDLINE. The search keywords were as follows: “melatonin”, “phytomelatonin”, “hypertension”, “blood pressure”, “pregnancy”, “mother”, “maternal”, “gestation”, “lactation”, “neonatal”, “perinatal”, “developmental programming”, “DOHaD”, “offspring”, “progeny”, and “reprogramming”. Relevant abstracts were identified and reviewed to identify suitable reports. Appropriate published articles in English-language journals were included, without restriction regarding the time of publication.

## 2. Effect of Melatonin

### 2.1. Metabolism, Structure, and Function

*N*-acetyl-5-methoxytryptamine, commonly known as melatonin, is an endogenously formed indoleamine comprising two functional groups, the 5-methoxy group and the N-acetyl side chain, which determine its specificity and amphiphilicity [19]. Importantly, the amphiphilic nature of melatonin allows it to easily cross the placenta to provide circadian signals from mother to fetus. Along with the pineal gland, many other organs can produce melatonin, such as the skin, gastrointestinal tract, retina, and bone marrow [2]. The precursor to melatonin is tryptophan. Melatonin biosynthesis starts with tryptophan and consists of four enzymatic steps, but at least six enzymes are known to be involved [5]. Within melatonin biosynthesis, serotonin N-acetyltransferase is the rate-limiting enzyme and serotonin is an essential intermediate [5]. Additionally, in plants, the main melatonin metabolite is 2-, 3-, and 6-hydroxy-melatonin [20].

The half-life of melatonin in the circulation is generally short, varying in the range of 30–60 min [2]. Its amphiphilic characteristic allows melatonin to easily diffuse and cross all membranes. Approximately 70% of melatonin is bound to albumin, and the remaining 30% diffuses to the surrounding tissues after release into the blood [21].

Melatonin is largely metabolized in the liver and kidneys by P450 monooxygenases, followed by conjugation of the resulting 6-sulfatoxy-melatonin to produce the main metabolite, 6-sulfatoxymelatonin, in the urine [1]. In addition, melatonin can be metabolized by non-enzymatic pathways. N1-acetyl-N2-formyl-5-methoxykynuramine (AFMK) and N1-acetyl-5-methoxykynuramine (AMK) are two major melatonin-derived kynuramines of cyclic 3-hydroxymelatonin [3]. Similar to melatonin, these metabolites serve as powerful antioxidants [1,3].

### 2.2. Biological and Physiological Functions of Melatonin

Melatonin is a sleep–wake cycle-regulating hormone. Its rhythmic secretion is mediated by the master clock in the suprachiasmatic nucleus (SCN). The functional diversity of the melatonin receptors (MTs) contributes to a wide range of biological processes. Thus far, three mammalian melatonin receptor subtypes have been identified. The sleep-promoting effect of melatonin is attributed to the activation of the MT1 receptor in the SCN [22], while the phase-shifting activity of melatonin is linked to the MT2 receptor [22]. Besides two G-coupled receptors, MT1 and MT2, the MT3 receptor has been identified as quinone reductase 2 [23]. Melatonin may also interact with the nuclear receptor, retinoid acid receptor (ROR)/retinoid Z receptor (RZR) [21].

Along with its role in circadian rhythms, melatonin has several important physiological functions, including stimulation of the immune response; regulation of mitochondrial biogenesis; control of pancreas function and intestinal motility; prevention of tumor progression; as well as anti-inflammatory, free radical scavenger, antioxidant, and immunosuppressive actions [1,2,3,4,5]. Therefore, synthetic melatonin has been introduced as a dietary supplement and its preventive and therapeutic effects are known for many diseases [6,24,25,26,27,28].

### 2.3. Melatonin in Gestation and Fetal Development

Of note is that melatonin is crucial in fetal development during gestation [4,29,30]. Maternal serum melatonin levels are increased meaningfully during gestation, with the highest levels at term, and they decrease abruptly after delivery [31]. Melatonin can easily cross the placenta and enter the fetal circulation [32]. Accordingly, maternal daily rhythms can be transferred to the fetus to synchronize fetal circadian rhythms [33].

Besides being generated by the pineal gland, melatonin can be produced by the placenta independently of the circadian clock [34]. Placental melatonin does not only act with the MT1 and MT2 receptors; it can directly scavenge free radicals to diminish placental oxidative damage as well [29,30]. In view of the fact that melatonin receptors exist in several fetal tissues, it is believed that melatonin contributes to fetal growth and development [35]. The pituitary concentration of the isotope-labeled melatonin-binding site is highest in the 20-day-old rat fetus [36]. Likewise, melatonin receptors exist in many areas of the fetal human brain [37]. Additionally, the disruption of the circadian clock genes of mother mice impairs organ development in the fetus [38]. All of the above findings support the notion that maternal melatonin is presumably involved in fetal organogenesis and circadian rhythms.

After birth, melatonin synthesis in the pineal gland is activated. Newborns do not have sufficient melatonin at night, although they have a developed pineal gland [39]. Hence, daily rhythmic melatonin secretion does not appear until 3–5 months of age in infants [39]. From this time, a detectable rhythmic melatonin level is observed, which coincides chronologically with the development of a normal circadian rhythm.

## 3. Developmental and Early Life Origins of Hypertension

### 3.1. Human Evidence

Multiple lines of human evidence indicate that early life insult stimuli are associated with the risk of hypertension later in life. The Dutch Hunger Winter cohort study is a famous example [40]. Offspring born to mothers exposed to famine during pregnancy develop many chronic diseases involving hypertension in later life [40,41,42].

The second line of evidence for reinforcement comes from investigation into suboptimal in utero conditions leading to preterm birth and low birth weight (LBW). Numerous epidemiological studies confirm that preterm birth and LBW are important risk factors for hypertension later in life [11,43]. Preterm birth or very LBW subjects have higher systolic BP in adulthood, which has been revealed in a meta-analysis recruiting 1342 preterm participants [11]. Third, several risk factors of the early origins of hypertension have been identified in mother–child cohorts. These risk factors include maternal undernutrition [41], maternal obesity [44], maternal smoking [45], gestational hypertension [46], short-term breastfeeding [47], maternal vitamin D deficiency [48], etc.

### 3.2. Animal Evidence

Although observational studies cannot be used to establish a cause–effect relationship between adverse early life environmental factors and hypertension in later life, increasing evidence from animal research has confirmed different types of early life insult stimuli that drive hypertension programming and identify potential mechanisms. Previous reviews indicate that maternal nutritional imbalances, maternal medical conditions, environmental exposure to chemicals, and maternal medication all can induce developmental programming resulting hypertension in adult offspring [10,11,12,13,49,50]. The most commonly used species are rats [13]. Other species such as sheep [51], cows [52], chicken [53], and mice [54] have also been used to assess hypertension of developmental origins.

A growing body of evidence suggests that there may be some core mechanisms involved in the pathogenesis of the early life origins of hypertension. Animal models have provided substantial insights into certain mechanisms, such as an aberrant renin–angiotensin system (RAS) [55], dysregulated nutrient-sensing signals [56], gut microbiota dysbiosis [57], oxidative stress [58], impaired NO signaling [59], epigenetic regulation [60], etc. Given that detailed reviews of each mechanism are beyond the scope of this paper, readers are referred elsewhere.

**Figure 1:**
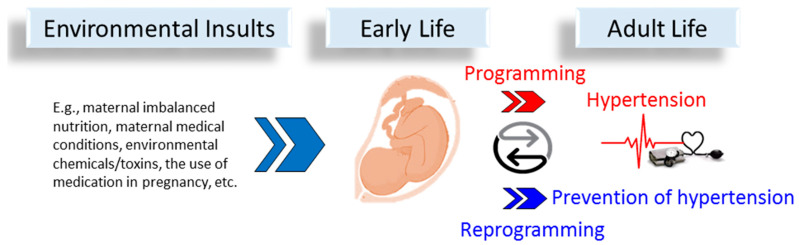
Schema outlining the environmental insults in early life that may induce developmental programming, leading to hypertension in adult life. Conversely, early reprogramming intervention could prevent the development of hypertension in later life.

Even though the complete mechanisms behind the early origins of hypertension remain inconclusive at present, our understanding of potential molecular mechanisms has advanced greatly in recent years through the use of animal models, which aid in developing efficient reprogramming interventions to prevent hypertension from happening (see Figure 1 for programming and reprogramming of early life origins of hypertension).

## 4. Melatonin Use in Current Clinical Practice

### 4.1. Dosage and Side Effects

In the United States, over-the-counter (OTC) melatonin is the second and fourth most popular nonvitamin and nonmineral dietary supplement taken by children and adults, respectively [61,62]. In contrast to its countrywide use as a dietary supplement in the United States, melatonin is available by prescription only in the European Union, United Kingdom, Japan, Australia, and Canada.

OTC melatonin is available in dosages from 1 mg to 10 mg in the United States. Melatonin can be taken as tablets, liquids, pills, cream, or sublingual drops. Most formulations are mainly composed of synthetic melatonin, while only a few sources are from plants [63]. Considering that oral melatonin has poor and variable bioavailability, the intranasal, transdermal, and oral transmucosal administration of melatonin have significant potential, although more human research is still warranted [64].

Oral melatonin supplementation in humans has been studied in a wide range of dosages from 0.3 mg to 1600 mg daily [65]. Thus far, the usual daily doses of melatonin range from 2 mg to 10 mg in diverse populations. No studies have reported that exogenous melatonin causes any serious adverse effects. The main reported adverse effects include fatigue, excessive sleepiness, and reductions in neurocognitive and psychomotor function [65].

### 4.2. Melatonin Use in Hypertension

Melatonin has been supplemented in the treatment of a broad spectrum of human diseases [9,66,67]. Its BP-lowering effects have been examined in several randomized controlled trials (RCTs). A meta-analysis study pooling five RCTs identified significant reductions for systolic BP (median (MD): −3.43 mmHg, 95% confidence interval (CI): −5.76 to −1.09) and diastolic BP (MD:  −3.33 mmHg, 95% CI:  −4.57 to −2.08), observed after melatonin supplement treatment relative to a placebo [68]. Melatonin was supplemented in doses ranging from 3 to 10 mg/day for 6–12 weeks. Likewise, another meta-analysis study recruiting eight RCTs revealed that melatonin supplementation significantly reduced systolic and diastolic BPs in patients with metabolic disorders [69]. Additionally, exogenous melatonin prescription has been reported with insignificant effects on nocturnal BP [70].

### 4.3. Melatonin Use in Pregnancy and Neonatal Diseases

Thus far, little reliable information exists regarding melatonin’s use and safety in pregnant or breastfeeding women based on available data from clinical trials. Although pregnant rats and sheep that received up to 10 times higher doses than the usual dose of melatonin during gestation displayed no adverse effect on fetal or maternal health [71,72], pregnant and lactating women are not recommended for melatonin use because of a lack of human studies [73].

Melatonin has been used in several neonatal diseases, including hypoxic–ischemic injury [74], respiratory distress syndrome [75], sepsis [76], and adjunct analgesic therapy [77]. Nevertheless, most studies have a very small sample size. Whether melatonin is an effective therapy in neonatal disorders awaits further evaluation. Similar to adults, melatonin has a generally favorable safety profile in the pediatric population [27,28]. There were very few significant adverse events being reported in children under melatonin treatment [78,79].

Overall, melatonin supplementation in humans appears to be relatively safe. However, further research into the use of melatonin in gestation and lactation in determining long-term offspring outcomes is urgently needed.

## 5. Melatonin Use for Early Life Origins of Hypertension

The DOHaD concept offers an opportunity to prevent the development of adult diseases during early stages of life by early therapeutic intervention. As such, adverse programming processes can be stopped or delayed before the clinical onset of the disease, known as reprogramming [15]. As melatonin has pleiotropically biological functions, the early use of melatonin in gestation and lactation may act as a reprogramming therapy to protect offspring against various adult diseases. Although there are several research studies documenting melatonin use in pregnancy and lactation, formal reviews indicate that only small parts of them are focused on offspring outcomes [18,80]. Here, we list in Table 1 a summary of studies documenting the reprogramming effects of melatonin in animal models regarding hypertension of developmental origins [81,82,83,84,85,86,87,88,89,90,91,92,93,94,95]. The therapeutic duration is only restricted to fetal and childhood stages before the onset of hypertension.

Rats are the most commonly used animal species. Rats become sexually mature at around six weeks old. In the adult rat, one month is approximately equivalent to three human years [96]. Hence, Table 1 lists the rat ages determined for reprogramming effects ranging from 8 to 27 weeks, which can be translated to the adolescent to young adult stages in humans. Thus far, there is a substantial lack of knowledge of the lifelong effects of melatonin in older adults.

Although maternal melatonin use has been examined for fetal programming in mice, sheep, and cattle [97,98], there are no data about the use of other animal species to evaluate the impact of melatonin in pregnancy and lactation on the early origins of hypertension.

Table 1 shows that various insult stimuli cause the developmental programming of hyper tension in adult offspring, which can be prevented by early melatonin treatment. These early life insults include a maternal high-fructose diet [86], maternal dietary restriction [87], maternal N^G^-nitro-L-arginine-methyl ester (L-NAME) exposure [88], maternal continuous light exposure [89], maternal methyl-donor diet [90], combined maternal high-fructose and postnatal high-salt diets [91], antenatal and neonatal dexamethasone exposure [92,93,94], and prenatal dexamethasone exposure plus post-weaning high-fat diet [95]. Additionally, early melatonin treatment has been tested in young rats using the spontaneously hypertensive rat (SHR) model [81,82,83,84] as well as the adenine-induced pediatric chronic kidney disease (CKD) model [85]. In general, melatonin is administered in drinking water during pregnancy and lactation according to most studies. Accordingly, the protective effects of melatonin on hypertension in these models are basically considered as reprogramming instead of direct effects.

### 5.1. Protective Mechanisms of Melatonin against Early Life Origins of Hypertension

**Figure 2:**
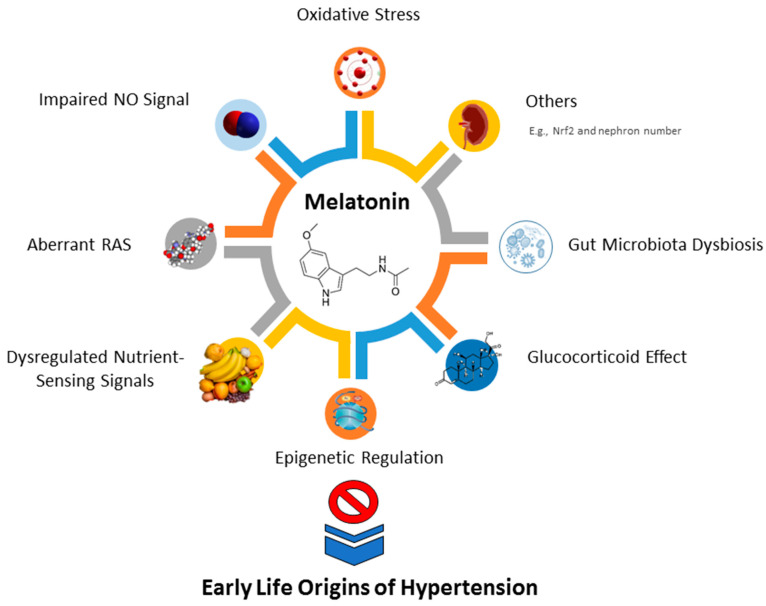
Schema outlining the protective mechanisms of early life melatonin use against early life origins of hypertension. NO = nitric oxide; Nrf2 = nuclear factor erythroid 2-related factor 2; RAS = renin–angiotensin system.

In view of the fact that different early life insult stimuli produce the same outcome―hypertension in later life—there might be some common mechanisms involved in the pathogenesis of the early life origins of hypertension. Notably, data from experimental animal studies have revealed an interplay between melatonin and the aforementioned mechanisms, such as oxidative stress, impaired NO signaling, aberrant RAS, dysregulated nutrient-sensing signals, epigenetic regulation, glucocorticoid effect, and gut microbiota dysbiosis. Figure 2 presents an illustration of the protective mechanisms of melatonin interlinked with the early life origins of hypertension. Each mechanism is discussed in turn.

### 5.2. Oxidative Stress

During pregnancy, the low levels of antioxidants in the fetus are inadequate to overcome excessive reactive oxygen species (ROS) under adverse environmental conditions, resulting in oxidative stress damage [99]. Melatonin is a well-known, potent antioxidant molecule against oxidative stress [2,3]. Melatonin is capable of scavenging ROS [100]; stimulating the gene expression of antioxidant enzymes [101]; and protecting lipids, proteins, and nuclear DNA from oxidative damage [2,3]. A previous review demonstrated that several early life insults linked the early life origins of hypertension to oxidative stress [58], including maternal nutritional imbalance, maternal illness, pregnancy complications, medication use in pregnancy, and exposure to environmental chemicals.

Prior studies support the protective effects of maternal melatonin therapy against oxidative stress-related early life origins of hypertension in models of a maternal high-fructose diet [86], maternal dietary restriction [87], maternal L-NAME exposure [88], maternal methyl-donor diet [90], and glucocorticoid exposure [92]. The main beneficial mechanisms underlying the actions of melatonin consisted of decreased ROS-producing enzyme expression, reduced ROS production, increased antioxidant capacity, and decreased oxidative DNA damage. However, one study showed that maternal melatonin treatment reduced the elevation of BP in 8-week-old male SHR offspring, which was not related to the activities of catalase, superoxide dismutase, and glutathione reductase [81]. In spite of recent advances in understanding how early life oxidative stress impacts the early life origins of hypertension, further research is required to elucidate the reprogramming mechanisms of melatonin, particularly regarding which developmental window and which organ-specific redox-sensitive signaling pathway are responsible for its beneficial effects.

### 5.3. Impaired NO Signaling

NO plays a pivotal role in the regulation of BP. Impaired NO signaling is a common mechanism behind the early life origins of hypertension, while NO-targeting interventions in early life may act as a reprogramming strategy to prevent the development of hypertension in adulthood [59]. NO is formed from the conversion of L-arginine to L-citrulline by nitric oxide synthase (NOS), while asymmetric dimethylarginine (ADMA) can compete with L-arginine for NOS, contributing to impaired NO signaling [102].

Melatonin can reduce ADMA to regulate NO [103]. SHRs exhibited a higher BP than control normotensive Wistar Kyoto (WKY) rats, which was related to increased plasma ADMA levels [82,83]. In young SHRs, the BP-lowering effect of melatonin was associated with an increase in renal dimethylarginine dimethylaminohydrolase (DDAH; ADMA-metabolizing enzymes) activity to decrease ADMA in the plasma and kidneys [82,83]. NO inhibition induced during gestation by the administration of L-NAME elevated offspring BP, which maternal melatonin therapy prevented [88]. Additionally, melatonin use in pregnancy and lactation was of benefit for offspring hypertension coinciding with the restoration of the ADMA-NO balance in several animal models related to the early life origins of hypertension [86,87,91]. In the pediatric CKD model [85], early melatonin therapy prevented CKD-induced hypertension and kidney damage, coinciding with a reduction in ADMA. These results support the notion that the NO signaling pathway may be a core mechanism behind the early life origins of hypertension and the reprogramming effects of melatonin.

### 5.4. Aberrant RAS

In addition to the regulation of BP, RAS is a notable hormonal cascade controlling kidney development [104,105]. The classic RAS can be defined as the angiotensin-converting enzyme (ACE)/Ang II/angiotensin type 1 receptor (AT1R) axis, which promotes vasoconstriction. Prior research suggests a transient biphasic response with downregulation of the classic RAS in the neonatal stage that becomes normalized with age. Early life insults can disturb this normalization and inappropriately activate the classic RAS axis, resulting in hypertension and kidney disease in adult offspring [55,106,107].

Accumulative evidence suggests that the interplay between melatonin and RAS determines BP [108]. In melatonin-deficient hypertension, the classic RAS axis is activated [109]. Conversely, activation of the classic RAS could be blocked by melatonin therapy, coinciding with the prevention of offspring hypertension, in a variety of animal models, including maternal high-fructose diet [86], maternal dietary restriction [87], and maternal continuous light exposure [89]. Besides the classic RAS axis, the non-classic ACE2/angiotensin (1–7)/Mas receptor axis has also been linked to the early life origins of hypertension [105]. A previous report indicated that maternal melatonin therapy prevents offspring hypertension, accompanied by increased mRNA expression of *Agtr1b* and *Mas1* in a model of combined prenatal dexamethasone exposure and high-fat diet [95]. Hence, these observations indicate a crosstalk between RAS and melatonin, by which both tightly mediate the developmental programming of hypertension.

### 5.5. Dysregulated Nutrient-Sensing Signals

Several nutrient-sensing signals are involved in the early life origins of hypertension, including AMP-activated protein kinase (AMPK), peroxisome proliferator-activated receptors (PPARs), silent information regulator T1 (SIRT1), and PPARγ co-activator 1α (PGC-1α) [110,111]. Nutrient-sensing signals orchestrate fetal metabolism in response to maternal nutritional status in pregnancy, while these signals can be disturbed by early life nutritional insults. SIRT1 and AMPK can, respectively, deacetylate and acetylate PGC-1α, to mediate PPARs and their target genes, thereby resulting in hypertension later in life [112,113].

Prior research indicates that melatonin’s benefits can be attributed to the activation of nutrient-sensing signals [114,115,116]. Likewise, melatonin’s protection of adult offspring against hypertension is associated with the activation of the AMPK/SIRT1/PGC-1α pathway. A maternal methyl-donor diet led to offspring hypertension accompanied by reduced renal expression of several nutrient-sensing signaling components, comprising AMPKα2, SIRT1, PPARβ, and PPARγ [90]. Another study showed that the use of melatonin in gestation and lactation prevented hypertension programmed by a combined high-fructose plus post-weaning high-salt diet via regulating SIRT1, SIRT4, AMPKα2, AMPKβ2, PPARγ, and PGC-1α in the kidneys [91]. These findings are consistent with previous studies showing that AMPK activation prevents offspring hypertension via regulating nutrient-sensing signals in various models of the early life origins of hypertension [111]. Although a link between nutrient-sensing signaling and melatonin has been established, we still do not know if these reprogramming effects of melatonin are organ-specific in the early life origins of hypertension.

### 5.6. Epigenetic Regulation

Epigenetic regulation is another core mechanism underlying the reprogramming effects of melatonin to prevent the early life origins of hypertension [60,104]. Notably, melatonin is involved in epigenetic regulation [117,118]. Epigenetic processes, including DNA methylation, histone modification, and microRNA (miRNA), are known to influence gene expression [119]. Former research revealed that melatonin can serve as an inhibitor of DNA methyltransferases (DNMT) or act as a histone deacetylase (HDAC) inhibitor [93,117]. In an antenatal dexamethasone exposure model, melatonin not only protected against hypertension but also restored the brain reelin mRNA expression levels by reducing DNMT1 expression [92,120]. Melatonin also reduced the binding of methyl-CpG binding protein 2 (MeCP2) and DNMT1 to the reelin promoter [120]. Another study revealed that melatonin and trichostatin A (a HDAC inhibitor) provide similar benefits for neonatal dexamethasone-induced programmed hypertension [93], suggesting a potential protective mechanism underlying HDAC inhibition by melatonin. Although melatonin can regulate the expression of certain miRNAs and their target genes [121], their impact in the early life origins of hypertension remains unclear.

Furthermore, melatonin programs alterations in the renal transcriptome and genes involved in the melatonin signaling pathway during kidney development [118]. Using the RNA next-generation sequencing method to analyze the renal transcriptome, a total of 455, 230, and 132 differentially expressed genes were identified in the kidneys of offspring rats born to melatonin-treated dams at the age of 1, 12, and 16 weeks, respectively. Among them, several genes involved in the biosynthesis of melatonin were significantly up-regulated, including aromatic L-amino acid decarboxylase, tryptophan hydroxylase 1, and N-acetylserotonin methyltransferase. Additionally, several melatonin receptors were up-regulated in the offspring kidneys, including MT2, RORα, and RORβ. Moreover, maternal melatonin therapy mediates several biological pathways during kidney development, including the PPAR signaling pathway, focal adhesion signaling, fatty acid metabolism, the wingless-int (Wnt) signaling pathway, the transforming growth factor (TGF)-β signaling pathway, and the erythroblastic leukemia viral oncogene (ErbB) signaling pathway [118]. These observations support the notion that melatonin can epigenetically regulate specific genes and pathways, by which it prevents programmed hypertension. Its long-term epigenetic changes in later life, however, remain to be elucidated.

### 5.7. Glucocorticoid Effect

Similar to melatonin, glucocorticoid is involved in circadian rhythms [122,123]. During pregnancy, maternal melatonin and glucocorticoid are able to cross the placenta. Hence, both can establish and entrain the fetal circadian clock [122]. There is a crosstalk between glucocorticoid and melatonin, by which both chronobiotics tightly mediate developmental programming processes: melatonin can downregulate glucocorticoid receptor expression [109], while MT receptors could be downregulated following glucocorticoid treatment [124].

Early life glucocorticoid exposure through excessive maternal corticosteroids (e.g., compromised pregnancies) or through exogenous administration (e.g., preterm birth) can impair the fetal hypothalamic–pituitary–adrenal (HPA) axis, resulting in adult diseases, namely glucocorticoid programming [124,125]. Conversely, early life melatonin therapy is beneficial for the prenatal or neonatal glucocorticoid-induced programming of hypertension [92,93,94,95].

Another study demonstrated that melatonin or melatonin receptor agonist agomelatine can prevent offspring hypertension programmed by maternal exposure to continuous light [89]. In spite of the beneficial effects of melatonin use in pregnancy and lactation that have been reported in several models of maternal chronodisruption [123], the interplay between glucocorticoid programming, circadian rhythms, and melatonin in the early life origins of hypertension awaits further clarification.

### 5.8. Gut Microbiota Dysbiosis

Lastly, one protective mechanism of melatonin against the early life origins of hypertension might be attributed to its ability to mediate the gut microbiota. Of note is that melatonin is one of the tryptophan-derived metabolites, and many metabolites are gut microbial catabolites regulated by the composition of the gut microbiota [126]. Along with the pineal gland, the gut is one of the main sources of melatonin, and its concentration in the gut is 10–100 times higher than that in the plasma [127]. The gut microbiota and its derived metabolites are involved in the regulation of BP [128,129]. These metabolites include short-chain fatty acids (SCFAs), trimethylamine-N-oxide (TMAO), tryptophan-derived uremic toxins, etc. [126,128,129]. Recent studies suggest that melatonin-mediated gut microbiota changes play a key role in certain diseases [130], although little is known about its impact in hypertension.

There is increasing evidence proposing that the gut microbiota in early life is linked to adult disease in later life [131]. Prior reviews reported a variety of early life insults that induce offspring hypertension related to alterations of the gut microbiota, while gut microbiota-targeted therapy, if applied early, can prevent hypertension in later life [57,132]. We recently found that early melatonin therapy protects young rats against CKD-induced hypertension and kidney damage [85]. The beneficial effects of melatonin include reshaping the gut microbiota, increased α-diversity, and enhancement of the abundance of the genus *Roseburia* and the phylum *Proteobacteria*. Additionally, melatonin reversed the changes to the plasma TMAO-to-TMA ratio induced by CKD in young rats of both sexes. However, the interaction mechanisms between melatonin and the gut microbiota underlying the early life origins of hypertension need to be further explored.

### 5.9. Others

Considering its pleiotropic bioactivities, there might be other mechanisms by which melatonin acts: (1) by activating Nrf2 (nuclear factor erythroid 2-related factor 2) and (2) by increasing the nephron number. Melatonin has been considered as an Nrf2 activator [133]. Nrf2 activation has shown benefits in several models of developmental hypertension [134,135,136]. However, this remains speculative, and the question of whether the reprogramming effects of melatonin with programmed hypertension occur directly through the regulation of Nrf2 deserves further evaluation.

In a prenatal dexamethasone exposure model [92], maternal melatonin therapy prevented offspring hypertension accompanied by the restoration of the nephron number programmed by glucocorticoid. As a reduced nephron number is a key mechanism behind the early life origins of hypertension [9], there might be an interplay between melatonin and mechanisms that determine the nephron number that leads to hypertension of developmental origins.

Although numerous mechanisms are outlined above, additional work is needed to explore other potential mechanisms. A deeper understanding of how melatonin reprograms hypertension in early life is key toward creating optimal interventions for its clinical translation.

## 6. Conclusions and Future Perspectives

Currently, there is general agreement that melatonin acts in various ways to benefit human health, with a favorable safety profile. Moreover, newly discovered evidence from animal studies on developmental programming suggests that the use of melatonin in early life could prevent the development of hypertension later in life.

There are still some questions that need to be addressed. Although there are clinical trials aiming to determine optimal doses and durations of melatonin to treat neonatal and fetal diseases, little reliable information regarding the effective dosage and therapeutic period of melatonin for pregnant women and its long-term effects on their offspring exists. As we summarize above, the protective effects of melatonin on the early life origins of hypertension can be attributed to several interaction mechanisms. Due to the multifaceted actions of melatonin, the reprogramming mechanisms of early life melatonin therapy might be complex and interrelated, and it is difficult to determine their relative importance. Further studies are required to take a holistic approach to the simultaneous determination of various reprogramming mechanisms in response to early melatonin use in one experiment.

Melatonin has a meaningful role in the prevention and control of hypertension. With a greater understanding of the early life origins of hypertension and tremendous growth in the early use of melatonin, we expect that melatonin as a reprogramming therapy will be applied in clinics to reduce the global burden of hypertension.

## Figures and Tables

**Table 1:** Reprogramming effects of melatonin protect against early origins of hypertension in animal models.

Melatonin Treatment	Period of Treatment	Animal Model	Species/ Gender	Age at Measurement	Reprogramming Effects	Ref.
Melatonin (10 mg/kg/day) in drinking water to dams	Pregnancy and lactation	Genetic hypertension	SHR/M	8 weeks	Decreases the rate of rise in BP	[81]
0.01% melatonin in drinking water to young rats	4–10 weeks of age	Genetic hypertension plus L-NAME exposure	SHR/M	10 weeks	Prevented hypertension, reduced oxidative stress and ADMA level in the kidneys	[82]
0.01% melatonin in drinking water to young rats	4–12 weeks of age	Genetic hypertension	SHR/M	12 weeks	Prevented hypertension, reduced oxidative stress and plasma ADMA level	[83]
Melatonin (20 μg/mL) in drinking water to dams	Pregnancy and lactation	Genetic hypertension	SHR/M	27 weeks	Prevented the rise in BP	[84]
Melatonin (10 mg/kg/day) in drinking water to young rats	3–6 weeks of age	Adenine-induced CKD	SD rat/M &F	9 weeks	Prevented hypertension, reversed the TMAO-to-TMA ratio, and restored gut microbiota alterations	[85]
0.01% melatonin in drinking water to dams	Pregnancy and lactation	Maternal high-fructose diet	SD rat/M	12 weeks	Prevented hypertension, altered renal transcriptome, and increased renal NO	[86]
0.01% melatonin in drinking water to dams	Pregnancy and lactation	Maternal dietary restriction	SD rat/M	12 weeks	Prevented hypertension, reduced plasma ADMA level, and increased renal NO	[87]
0.01% melatonin in drinking water to dams	Pregnancy and lactation	Maternal L-NAME exposure	SD rat/M	12 weeks	Prevented hypertension, altered renal transcriptome, and increased renal NO	[88]
0.01% melatonin in drinking water to dams	Pregnancy and lactation	Maternal continuous light exposure	SD rat/M	12 weeks	Prevented hypertension	[89]
0.01% melatonin in drinking water to dams	Pregnancy and lactation	Maternal high methyl-donor diet	SD rat/M	12 weeks	Attenuated hypertension and altered renal transcriptome	[90]
0.01% melatonin in drinking water to dams	Pregnancy and lactation	Maternal high-fructose diet plus post-weaning high-salt diet	SD rat/M	12 weeks	Attenuated hypertension and restored NO system	[91]
0.01% melatonin in drinking water to dams	Pregnancy and lactation	Prenatal dexamethasone exposure	SD rat/M	16 weeks	Prevented hypertension and reversed the reduction in nephron number	[92]
0.01% melatonin in drinking water to dams	Pregnancy and lactation	Neonatal dexamethasone exposure	SD rat/M	16 weeks	Prevented hypertension and preserved histone deacetylase gene expression	[93]
0.01% melatonin in drinking water to dams	Lactation	Neonatal dexamethasone exposure	SD rat/M	16 weeks	Prevented hypertension and increased renal melatonin level and MT2 protein	[94]
0.01% melatonin in drinking water to dams	Pregnancy and lactation	Prenatal dexamethasone exposure plus post-weaning high-fat diet	SD rat/M	16 weeks	Prevented hypertension and restored the aberrant RAS	[95]

SD rat = Sprague Dawley rat; M = male; F = female; ADMA = asymmetric dimethylarginine; TMAO = trimethylamine-N-oxide; TMA = trimethylamine; NO = nitric oxide; MT2 = melatonin receptor 2; CKD = chronic kidney disease; L-NAME = N^G^-nitro-l-arginine methyl ester; RAS = renin–angiotensin system.

## Data Availability

The data are contained within the article.
